# Author Correction: A physical wiring diagram for the human immune system

**DOI:** 10.1038/s41586-024-07928-6

**Published:** 2024-10-21

**Authors:** Jarrod Shilts, Yannik Severin, Francis Galaway, Nicole Müller-Sienerth, Zheng-Shan Chong, Sophie Pritchard, Sarah Teichmann, Roser Vento-Tormo, Berend Snijder, Gavin J. Wright

**Affiliations:** 1 Cell Surface Signalling Laboratory, Wellcome Sanger Institute, Cambridge, UK; 2 Institute of Molecular Systems Biology, ETH Zurich, Zurich, Switzerland; 3 Cellular Genetics Programme, Wellcome Sanger Institute, Cambridge, UK; 4 Department of Biology, Hull York Medical School, York Biomedical Research Institute, University of York, York, UK

**Keywords:** Immunochemistry, Multicellular systems

Correction to: *Nature* 10.1038/s41586-022-05028-x Published online 3 August 2022

In the version of the article initially published, “CLC4M” was missing from Fig. 1e and “20 nM” originally read “20 µm” in Fig. 2c. Additionally, the first sentence of the “Processing of single-cell RNA-sequencing data” section of the Methods was missing four references which have now been added as refs. 67–70: Park, J.-E. et al. A cell atlas of human thymic development defines T cell repertoire formation. *Science* **367**, eaay3224 (2020); Vieira Braga, F. A. et al. A cellular census of human lungs identifies novel cell states in health and in asthma. *Nat. Med*. **25**, 1153–1163 (2019); Young, M. D. et al. Single-cell transcriptomes from human kidneys reveal the cellular identity of renal tumors. *Science* **361**, 594–599 (2018) and Madissoon, E. et al. scRNA-seq assessment of the human lung, spleen, and esophagus tissue stability after cold preservation. *Genome Biol*. **21**, 1 (2019). These corrections have been made to the HTML and PDF versions of the article.

