# Supplementary material for: A physical wiring diagram for the human immune system
Source: Nature. 2022 Aug 3;608(7922):397–404. doi: 10.1038/s41586-022-05028-x (PMC9365698; doi:10.1038/s41586-022-05028-x)
Supplement: Supplementary file 1 — Supplementary Equations - a technical overview of the mathematical models implemented, along with derivations and definitions of equations. The text begins with a summary overview before defining modelling assumptions and calculations in detail. [file 41586_2022_5028_MOESM1_ESM.pdf]

nature portfolio

https://doi.org/10.1038/s41586-022-05028-x

Supplementary information

A physical wiring diagram for the human immune system

In the format provided by the authors and unedited

Mechanistic model of immune cell interactions

Shilts *et al.*

April 19, 2022

Abstract

The interactions between immune cells circulating through the blood allow the immune system to react to stimuli and maintain tolerance against autoimmunity. We have conducted the first systematic survey of all the physical interactions that take place between the surface proteins on human immune cells, and quantified the kinetics of the interactions we discovered to establish a detailed quantitative map of cellular interactions. Combining this map with a dataset of absolute proteomics quantification of the cell-surface proteins expressed on different immune cell types, we develop a "ground-up" model of the contacts made between human immune cells, based on exhaustively modeling all the binding events between cell populations.

Conceptual Overview

The intuition behind this model is a relatively simple hypothesis : if cell-to-cell adhesion is mostly determined by the binding of complementary receptors on the surfaces of those cells, and a relatively complete listing of these receptors has now been measured, then it should be possible to predict which cells are most likely to physically interact with each other by counting up their number of binding receptors. The number of receptors that would be bound between the membranes of two cells would reflect three processes:

– Out of the proteins on the surfaces of the two cells, how many are complementary and can bind each other? Pairs of cells with more complementary receptors and ligands would be more likely to physically interact than pairs of cells with few.

– For those binding receptors, how highly expressed are they? Pairs of cells with abundant levels of a surface protein involved in a binding interactions would be more likely to physically interact than pairs of cells with low levels.

– For each binding interaction between two cell-surface receptors, how strong is the interaction? Pairs of cells with higher-affinity interactions would be more likely to physically interact than pairs of cells with comparatively low-affinity interactions.

These different factors of course need to be integrated to make reliable predictions about the propensity of two cells to interact with each other. It is essential to quantiatively model these factors in the physically-realistic way, because simple heuristic 'rules' would otherwise fail to resolve ambiguous situations such as answering how likely two cells are to interact if they have high expression of a low-affinity receptor interaction compared to if they have low expression of a high-affinity receptor.

Our approach is to use equations derived from the law of mass action to provide a simple yet biophysically-grounded method of quantitatively aggregating these different factors into a final prediction of cellular interconnectivity. The concentrations of surface proteins (e.g. [▲], [⌄]) can be combined with the binding affinity (expressed as a dissociation constant, K_{D}) to calculate a hypothetical quantity of bound receptors between the cells if they reached a perfect equilibrium. Summing those quantities of bound proteins for a cellular pair then provides a basis for inferring cellular interconnectivity.

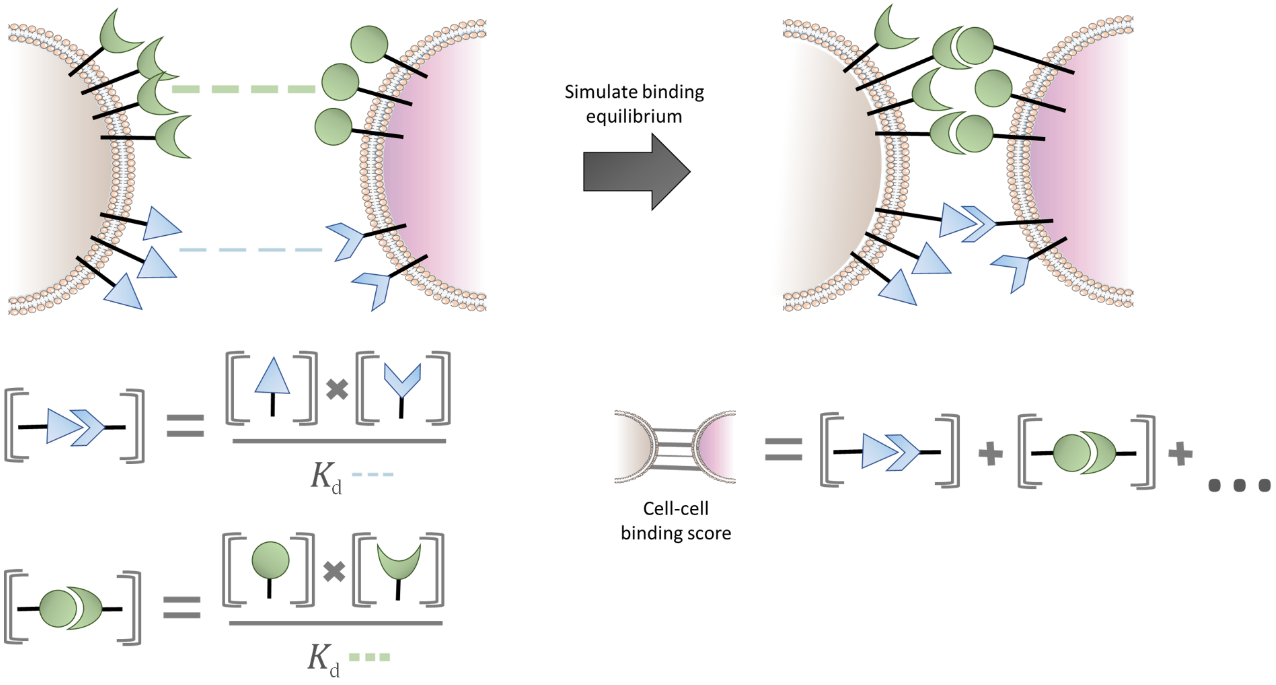

Although the cell-cell binding scores generated by the model are not the exact quantities that would be seen *in vivo* given assumptions described in the next section, they are nevertheless very useful predictors of cellular behavior in the context of multicellular communities like human immune cells from circulation.

As a concluding illustration of the power of these quantitative modeling approaching, consider a system made up of 3 distinct cell types that are mixed together. At the simplest level, one could add-up the number of distinct interacting protein pairs between each cell pair. Although simple, the basic premise of this has proven successful in other contexts, such as in a previous analysis that counted the number of distinct receptor binding pairs detected between two cell types and found that could correlate with the cell's co-occurrence in spatial images of a tissue [83]. However, those successes could be due to a backwards causality: cells physically proximal in a tissue would be likely to evolve more complementary receptors to communicate. As depicted in the schematic below, often in contexts like the immune system this rudimentary counting approach will either fail to detect substantial differences between cell pairs or produce misleading estimates due to counting the numerous receptors which have little functional role in adhesion.

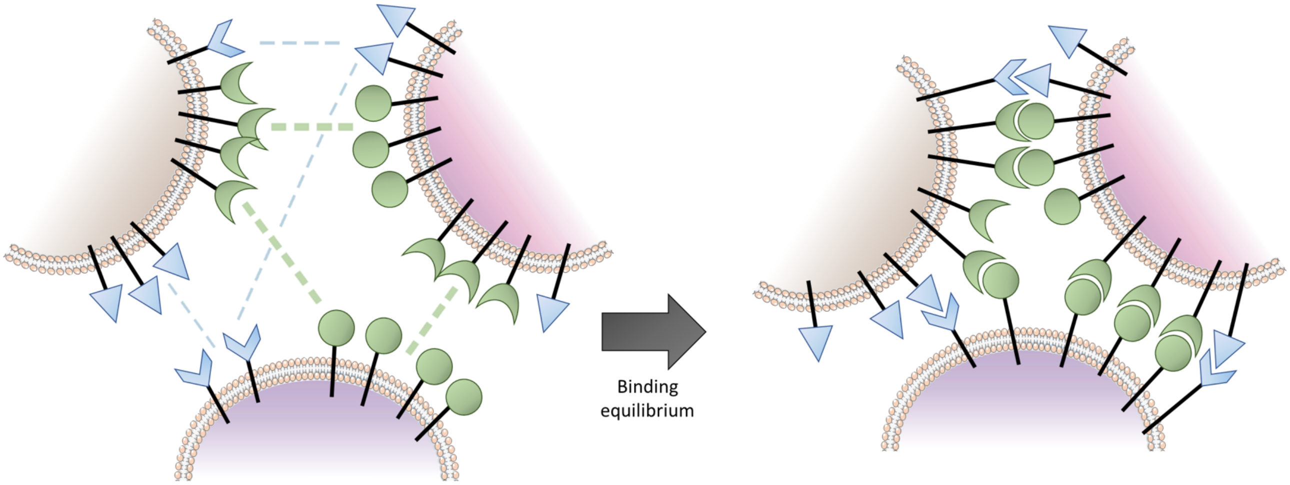

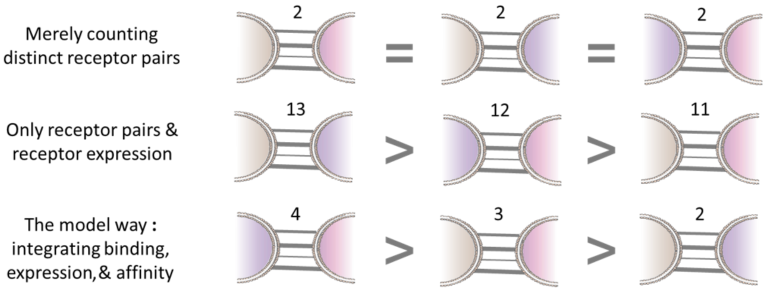

Similarly, as mentioned before, only considering one variable at a time, such as constructing a model purely based on interacting proteins and their corresponding expression levels, can be misleading because it neglects the substantial role that quantitative binding strength plays in determining which protein interactions are major contributors to cellular adhesion.

With an intuition for the conceptual basis of the model now described, in the next sections the technical features and derivation of the model will be explained in detail.

Core model

The core model integrates information about molecular interactions, their affinities, and expression levels in order to predict relative 'interaction scores' that represent the fundamental tendency of two cells to physically interact with one another. This reductionist model uses the equations of mass action kinetics to make principled inferences on cellular connectivity. It is important to note that many layers of complexity beyond this can influence intercellular interactions [85], but the formulation of this model provides a useful intermediate-scale representation of the system without demanding prohibitive molecular dynamics calculations or others that are common for microscale representations.

1 Input datasets

1.1 Cell size measurements

Average values for cell sizes were taken from the BioNumbers database [90]. Cell radius measurements are used in cell geometry calculations, to adjust for size differences affecting receptor densities. It is worth noting that we found the most accurate cell geometry values available result in the total surface-protein expression levels between cell types to become roughly normalized, so no cell type has broadly higher surface expression densities than the others. This criteria of eliminating biases can be used when multiple values are available in the literature.

1.2 Protein expression data

A previous study used high-sensitivity mass spectrometry proteomics to measure all the proteins expressed by purified human immune cell types [1]. Their data were calibrated against a standardized "ruler" so each measurement represents an absolute count of protein copy-number per cell. Measurements were taken for 4 human donors to provide estimates of expression variability.

1.3 Cell type fractions

In most comparisons between the model and experimental data, the cell type labels available in the proteomics do not perfectly align with the labels observed in the experiment. Therefore where needed we incorporated data of the fraction of each cell subtype that comprises a larger cell type category (e.g. if total CD14+ monocytes is the experimentally-observed category, while the proteomics measures classical, nonclassical, and intermediate CD14+ monocytes, then the fraction of those 3 monocyte subtypes is needed to approximate the measured total-monocyte category). Datasets include [89] (CD4 T cells), [91] (Helper CD4 T cells), [93] (Natural killer cells), [94] (CD8 T cells), [92] (Dendritic cells), [86] (Monocytes), [84] (Regulatory T cells), [95] (Regulatory T cells), and [87] (B cells).

1.4 Binary interaction network

We completed a systematic binary interaction screen of 630 immune cell surface proteins, covering every cell-surface protein detected in the proteomics dataset across all cell types and states (except for those such as multi-pass or highly polymorphic proteins which we are not able to express), plus proteins assigned a "CD" number that were missed by the proteomics. We validated our new interaction hits with an independent secondary screen and surface plasmon resonance (SPR). The list of new interactions we validated and all previously-published interactions validated by a high-quality method such as isothermal titration calorimetry, analytical ultracentrifugation, or SPR combined to make our final interaction network.

1.5 Binding kinetics

For every new interaction we discovered, we measured its binding with SPR and fit a model curve to calculate its equilibrium dissociation constant (K_{D}). For all of the previously-published interactions in our network, we found published kinetic data with reported K_{D} values.

2 Mass action equations

From the Law of Mass Action, a second-order chemical reaction such as two protein species, denoted **A** and **B**, binding to form complex **AB** can be described in terms of its equilibrium K_{D} in 3 dimensions

$$K_D^{(3D)} = \frac{k_{off}}{k_{on}} = \frac{[A] \cdot [B]}{[AB]} \tag{1}$$

While these equations were derived based on the statistics of random collisions in 3 dimensions, in the context of cell-to-cell interactions, the proteins on the surface of each cell are bounded to diffuse in only 2 dimensions. To approximate this conversion, the 3-dimensional K_{D} (${K_{\mathrm{D}}}^{\mathrm{3D}}$) can be multiplied by a "confinement length", h, representing the average height dimension of proteins on the cell membrane to give the 2-dimensional K_{D} (${K_{\mathrm{D}}}^{\mathrm{2D}}$) [97]. The value of h can be roughly estimated as 10 nm, although empirical measurements exist for several specific surface proteins.

$$K_D^{(2D)} = h \cdot K_D^{(3D)} \tag{2}$$

The proteomics data measures total levels of **A** and **B**, while the equations above describe the concentrations of bound and unbound molecules. Because cell volume is treated as a constant and binding reactions do not destroy or create proteins, the conservation of molecules can be invoked to determine the total protein levels measured.

$$[A_{total}] = [A] + [AB] \tag{3}$$
$$[B_{total}] = [B] + [AB] \tag{4}$$

While the equation above was defined based on the 3D concentrations of molecules (denoted [A]) measured when ${K_\mathrm{D}}^{3\mathrm{D}}$ is calculated by SPR, the same relationship applies to 2D concentrations of molecules (denoted [[A]]). Rewriting the 3-dimensional mass-action equation in terms of the 2-dimensional proteomic measurements yields

$$\begin{aligned} h \cdot K_D^{(3D)} &= \frac{[[A]] \cdot [[B]]}{[[AB]]} \\ &= \frac{([[A_{total}]] - [[AB]]) \cdot ([[B_{total}]] - [[AB]])}{[[AB]]} \\ &= (\frac{[[A_{total}]] \cdot [[B_{total}]]}{[[AB]]} - [[B_{total}]] - [[A_{total}]] + [[AB]]) \end{aligned} \tag{5}$$

This equation can be re-arranged to derive a formula for the fraction of bound proteins given the total protein amounts. First terms are grouped to give a quadratic expression with respect to the desired output, the bound concentration [[AB]].

$$[[AB]]^2 - ([[B]] + [[A]] + h \cdot K_D^{(3D)}) \cdot [[AB]] + [[A]] \cdot [[B]] = 0 \tag{6}$$

Only the negative root of the quadratic is considered because the positive root fails to satisfy the inequalities (3) and (4). Replacing the two-dimensional dissociation constant with the easier to measure ${K_\mathrm{D}}^{3\mathrm{D}}$ using the approximation in (2), this yields a final equation entirely in terms that we can experimentally measure.

$$\begin{aligned} [[AB]] = & -0.5 \cdot (\sqrt{([[A_{total}]] + [[B_{total}]] + h \cdot K_D^{(3D)})^2 - (4 \cdot [[A_{total}]] \cdot [[B_{total}]])} \\ & + [[A_{total}]] + [[B_{total}]] + h \cdot K_D^{(3D)}) \end{aligned} \tag{7}$$

These equations assume that **A** and **B** only participate in one interaction out of all immune surface proteins, which is true for most but not all proteins. In the future it could be possible to derive equations that factor in multiple equilibria between binding reactions involving a protein (or proteins) in common.

3 Cell interaction model

3.1 Assumptions

To predict the frequencies at which different cells interact, our model makes the following assumptions :

(i) Immune cells that are in circulation are well-mixed and do not have a fixed spatial structure. Because of this, the odds of any two cell types encountering each other is equal before an equilibrium is reached based on the cell's surface molecules which we model.

(ii) Cell-surface proteins on each cell are evenly dispersed overall. The probability of a given surface protein species on a cell physically encountering another molecule is proportional the density of that species, and not substantially changed on average by, for example, all the available proteins clustering on one small region of the cell membrane.

(iii) The strength of multiple interactions is the sum of the constituent interactions. The kinetic equations used in our model do not factor in how avidity may make the combined affinity of multiple to scale nonlinearly compared to the sum of those individual interactions.

(iv) Our interaction network is complete enough that it can describe the global interaction patterns of cells, even though no interaction network is likely to be cover every single molecular interaction that can occur between cells, including those not mediated by proteins.

(v) The simplified geometry of our model is sufficient to describe the complex interactions of cells. This includes our conversion of kinetic parameters to 2-dimensions based on an average confinement length and averaging out the shape of cells to be roughly spherical for calculations.

Given the immense additional complexity possible within areas such as micro-scale cell geometry and surface-protein clustering dynamics, we believe our model offers one of the most complete descriptions of aggregate-level system behavior possible without resorting to prohibitively large molecular dynamics simulations.

3.2 Model overview

First, for each cell type we defined an expression matrix of the copy numbers of every surface protein involved in a cell-to-cell interaction according to our systematic interaction network. The absolute copy numbers per cell were converted to 2-dimensional densities by assuming all cells are approximately spherical with a radius provided from the literature. For every possible pairing of cell types (including the cell type with itself), we repeated the following procedure: for all protein-protein interactions between the cell types, use equation (6) to calculate the concentration of protein expected to be bound in complex when the cells are in physical proximity.

$$A = 4 \cdot \pi \cdot r^2 \tag{8}$$

$$[[Molecules\ per\ area]] = \frac{molecule\ count\ per\ cell}{A} \tag{9}$$

We sum these bound concentrations together to derive a weighted interaction propensity score, I, specific to the cell-cell pair. When this procedure is done across all possible cell-cell pairs, the result is a symmetric matrix of relative cell affinities. From the relative affinities, it should be possible to predict how the occurrence of different cell interactions change in different conditions, such as upon a change in expression or pharmaceutical blocking of a particular receptor.

$$I_{x,y} = \sum_{i=1}^{n} [[AB_i]] \qquad \text{for n interactions between cells x and y} \tag{10}$$

This interaction score provides a relative measure of cellular connectivity. In units of molecules per surface area, it can be thought of conceptually as the quantity of bound molecules holding two cells together that would be present if all the surface proteins of those cells could perfectly equilibrate and access each other. Interaction scores can be directly correlated against observed rates of cellular association, to verify the computed scores correlate with observed interaction frequencies. This interaction score is also used as input for the differential equation extension to our core model, described later.

3.3 Validation

To calibrate and validate our model, we have access to high-throughput microscopy data of human immune cells that were mixed and labeled based on cell type [24]. The frequencies at which different cells contact each other is quantified, giving an experimental proxy for our interaction metrics.

We can correlate the measurements of how often different immune cells associate at equilibrium to model predictions. Because this core model does not include data on cell abundances, the observed frequencies would need to be adjusted against cell abundance. Otherwise, the most frequently-counted cellular interactions might simply be among cells that are more abundant, as opposed to having interaction rates above the baseline expectation. Scores for achieving that kind of normalization on experimental data have already been established [96].

Another direct test of the predictive power of this model would be to experimentally block specific cell-surface protein interactions (such as by adding recombinant ligands that competitively inhibit their receptors' interactions) and gauge how cell-to-cell contact frequencies change. If these changes are predictable from our "ground-up" mass action kinetics model, that would demonstrate the utility of our systematic approach. We describe these later in our differential equation model extension.

Future extensions of this model could also be interesting for spatial measurements of cells in tissues. Methods such as CODEX can label cell-type markers

on cells resident in tissues, and have been used to generate datasets counting the contact frequencies of immune cells in lymphoid tissues [88]. The relative interaction scores would provide predictions about how expression changes could alter cellular contacts.

References

[83] Baccin, C.; Al-Sabah, J.; Velten, L.; Helbling, P.; Grunschlager, F.; Hernandez-Malmierca, P.; Nombela-Arrieta, C.; Steinmetz, L.; Trumpp, A.; Haas, S. (2020) Combined single-cell and spatial transcriptomics reveal the molecular, cellular and spatial bone marrow niche organization. Nature Cell Biology 22 (1), 38-48.

[84] Baatar, D.; Olkhanud, P.; Sumitomo, K.; Taub, D.; Gress, R.; Biragyn, A. (2007). *Human Peripheral Blood T Regulatory Cells (Tregs), Functionally Primed CCR4+ Tregs and Unprimed CCR4- Tregs, Regulate Effector T Cells Using FasL.* Journal of Immunology 178 (8), 4891–4900.

[85] Belardi, B.; Son, S.; Felce, J.; Dustin, M.; Fletcher, D. (2020). *Cell-Cell Interfaces as Specialized Compartments Directing Cell Function.* Nature Reviews Molecular Cell Biology 21 (12) 750–64.

[86] Boyette, L. B.; Macedo, C.; Hadi, K.; Elinoff, B. D.; Walters, J. T.; Ramaswami, B.; Chalasani, G.; Taboas, J. M.; Lakkis, F. G.; Metes, D. M. (2017). *Phenotype, Function, and Differentiation Potential of Human Monocyte Subsets.* PLoS One 12 (4), e0176460.

[87] Caraux, A.; Klein, B.; Paiva, B.; Bret, C.; Schmitz, A.; Fuhler, G. M.; Bos, N. A.; Johnsen, H. E.; Orfao, A.; Perez-Andres, M. (2010). *Circulating Human B and Plasma Cells. Age-Associated Changes in Counts and Detailed Characterization of Circulating Normal CD138- and CD138+ Plasma Cells.* Haematologica 95 (6), 1016–1020.

[88] Goltsev, Y.; Samusik, N.; Kennedy-Darling, K.; Bhate, S.; Hale, M.; Vazquez, G.; Black, S.; Nolan, G. (2018). *Deep profiling of mouse splenic architecture with CODEX multiplexed imaging.* Cell 174, 1-14.

[89] Kim, C. H.; Rott, L.; Kunkel, E. J.; Genovese, M. C.; Andrew, D. P.; Wu, L.; Butcher, E. C. (2001). *Rules of Chemokine Receptor Association with T Cell Polarization in Vivo.* Journal of Clinical Investigation 108 (9), 1331–1339.

[90] Milo, R.; Jorgensen, P.; Moran, U.; Weber, G.; Springer, M. (2010). *BioNumbers—the database of key numbers in molecular and cell biology.* Nucleic Acids Research 38, D750–D753.

[91] Niu, H.-Q.; Zhao, X.-C.; Li, W.; Xie, J.-F.; Liu, X.-Q.; Luo, J.; Zhao, W.-P.; Li, X.-F (2020). *Characteristics and Reference Ranges of CD4+T*

Cell Subpopulations among Healthy Adult Han Chinese in Shanxi Province, North China. BMC Immunology 21 (1), 44.

[92] Orsini, G.; Legitimo, A.; Failli, A.; Massei, F.; Biver, P.; Consolini, R. (2012). *Enumeration of Human Peripheral Blood Dendritic Cells throughout the Life*. International Immunology 24 (6), 347–356.

[93] Poli, A.; Michel, T.; Thérésine, M.; Andrès, E.; Hentges, F.; Zimmer, J. (2009). *CD56bright Natural Killer (NK) Cells: An Important NK Cell Subset*. Immunology 126 (4), 458–465.

[1] Rieckmann, J.C.; Geiger, R.; Hornburg, D.; Wolf, T.; Kveler, K.; Jarrossay, D.; Sallusto, F.; Shen-Orr, S.S.; Lanzavecchia, A.; Mann, M.; et al. (2017). *Social network architecture of human immune cells unveiled by quantitative proteomics*. Nature Immunology 18, 583–593.

[94] Romero, P.; Zippelius, A.; Kurth, I.; Pittet, M. J.; Touvrey, C.; Iancu, E. M.; Corthesy, P.; Devevre, E.; Speiser, D. E.; Rufer, N. (2007). *Four Functionally Distinct Populations of Human Effector-Memory CD8+ T Lymphocytes*. Journal of Immunology 178 (7), 4112–4119.

[95] Santner-Nanan, B.; Seddiki, N.; Zhu, E.; Quent, V.; Kelleher, A.; Fazekas de St Groth, B.; Nanan, R. (2008). *Accelerated Age-Dependent Transition of Human Regulatory T Cells to Effector Memory Phenotype*. International Immunology 20 (3), 375–383.

[96] Snijder, B.; Vladimer, G.; Krall, N.; Miura, K.; Schmolke AS.; Kornauth, C.; et al.(2017). *Image-based ex-vivo drug screening for patients with aggressive haematological malignancies: interim results from a single-arm, open-label, pilot study.*. The Lancet Haematology 4 (12), 595–606.

[97] Wu, Y.; Vendome, J.; Shapiro, L.; Ben-Shaul, A.; Honig, B. (2011). *Transforming binding affinities from three dimensions to two with application to cadherin clustering*. Nature 475, 510–513.

[24] Vladimer, G.I.; Snijder, B.; Krall, N.; Bigenzahn, J.W.; Huber, K.V.M.; Lardeau, C.-H.; Sanjiv, K.; Ringler, A.; Berglund, U.W.; Sabler, M.; et al. (2017). *Global survey of the immunomodulatory potential of common drugs*. Nature Chemical Biology 13, 681–690.

Differential equation extension

Although the core model can calculate the relative affinities for different immune cell populations, a limitation of these values is that they cannot effectively predict how perturbations alter immune cell interactions. For example, simulating any knockout of a receptor would only lead to predictions of cellular interactions decreasing, even though it would be expected that decreases in certain cellular interactions would accompany others that increase in frequency as cells are freed up from their original binding partners. Thus we complemented our core model with a differential equation extension that explicitly models all possible cell pairs associating and dissociating from each other with rate constants informed by our core model. This enables more accurate predictions of a range of perturbation conditions.

1 Input datasets

1.1 Cell proportions

The relative proportion of each immune cell type in the model in human blood was estimated from the BioNumbers database [90] and other published references [100] [98] [99].

1.2 Relative cell association rates

The mass-action rate constants for determining how a cell pair x and y associates and dissociate were directly taken from the $I_{x,y}$ values of the core model described above. Because the model gives greater scores for cell pairs with more predilection to associate, the inverse of these values was taken to give dissociation rate constants.

2 Cell-cell binding reactions

The law of mass action describes how reactants in a process equilibrate with their products. For example, formulas derived from mass action laws are what we previously used to formalize the reaction where a pair of proteins go from an unbound state to reversibly forming a bound complex. We reasoned the same basic principle could be applied to describe how cells in a mixture reversibly associate with each other in well-mixed equilibrium conditions. For blood immune cells in particular this analogy seems reasonable, given the high densities of immune cells (e.g. approximately 2.5 million per mL of blood) and their dynamic motility (in contrast to more static body tissues).

By this formulation, every cell type has the ability to bind with every other cell type. Thus the number of binding terms to account for increases combinatorially with the number of unique cell states (by the function $\binom{n}{2}$). For this

reason, we utilized rule-based algorithms to automatically construct the full system of differential equations for arbitrarily large models of cells [74].

3 Differential equation model details

3.1 Assumptions

Unlike the core mechanistic model that generates relative interaction scores, this extension of the model abstracts aspects of cell to cell binding in order to better estimate how cell interactions change under perturbations :

(i) All immune cells begin unbound, then reach an equilibrium after which frequencies of bound cell-cell pairs and unbound cells are constant. The time units in the model are arbitrary, so simulations proceed until equilibria are reached.

(ii) The relative interaction score from the mass-action model represents the dissociation constant for a particular pair of cells binding in a reversible reaction. The equilibrium constant for this reaction is treated as comprising a constant on-rate (k_{on}) for cells colliding into a bound cell-cell pair, and an off-rate (k_{off}) inversely proportional to the relative interaction score. The proportionality constant was selected to give approximately the expected fraction of cells in paired states versus singlet cells.

(iii) Cells are either in a single (unbound) state or a paired bound state. Higher-order combinations like 3 or more mutually bound cells are considered to occur at comparatively low rates and ignored.

3.2 Model extension overview

For brevity of illustration considering all equations share similar forms, one example each of differential equation type is given below. The first is for calculating the change in the proportion of a cell in its unbound state, and the other is for calculating the change in a cell in its paired state bound onto another cell. For a cell pair (e.g. CD4 T cells associating with dendritic cells):

$$\begin{aligned} T4 : DC'(t) = T4(t) \cdot DC(t) \cdot k_{on} \\ - T4 : DC(t) \cdot k_{T4:DC} \end{aligned} \tag{11}$$

While for the quantity of cells in an unbound state in a simple 5 cell type system (e.g. for calculating unbound CD4 T cells in a model also including B cells, NK cells, monocytes, and dendritic cells):

$$
\begin{aligned}
T4'(t) = {} & T4:NK(t)\cdot k_{T4:NK} + T4:DC(t)\cdot k_{T4:DC} \\
& + T4:M14(t)\cdot k_{T4:M14} + T4:B(t)\cdot k_{T4:B} \\
& + T4:T4(t)\cdot k_{T4:T4}\cdot 2 - 0.5\cdot T4(t)^2\cdot k_{on}\cdot 2 \\
& - T4(t)\cdot NK(t)\cdot k_{on} - T4(t)\cdot DC(t)\cdot k_{on} \\
& - T4(t)\cdot M14(t)\cdot k_{on} - T4(t)\cdot B(t)\cdot k_{on}
\end{aligned} \tag{12}
$$

Full systems of differential equations for a larger 7 cell-type model are provided in the appendix.

The ordinary differential equation system is then numerically solved from an initial condition corresponding to all cells being unbound at their natural proportions. In all simulations, a stable equilibrium is soon reached. At equilibrium, the proportion of every possible cell state (unbound, and all cell-cell combinations) is taken for further analysis. When simulating changes induced upon perturbation, the simulation is simply repeated after changing an input parameter.

3.3 Validation

The same automated microscopy methods used for validating the core model can also be adapted to testing how well the differential equation extension to the core model captures the effects of perturbations on cellular interactions. We conducted the first experimental tests pairing that automated blood immune cell imaging platform with recombinant proteins. Exogenously applying these soluble proteins which have binding activity to receptors on the surfaces of leukocytes can then act as perturbations. Although a rudimentary expectation would be that all soluble proteins act to disrupt the receptor's natural function of physically linking together cells, it is also conceivable in some cases that the soluble proteins could cluster the receptor in a way that does not block its binding sites, thus enhancing rather than disrupting an interaction.

Tests of more diverse kinds of perturbations in the future could also be possible by this same approach. For example, cells could have their expression profile significantly altered (such as by mutation or pre-stimulation). If the expression changes were precisely quantified, then the model predictions could be compared between using different expression matrices as inputs. Later improvements to the model could also be informative to determine how sensitively changes can be detected, such as would be needed to address more subtle questions like comparing receptor variants with known differences in affinity.

References

[98] Angelo, L. S.; Banerjee, P. P.; Monaco-Shawver, L.; Rosen, J. B.; Makedonas, G.; Forbes, L. R.; Mace, E. M.; Orange, J. S. (2015) *Practical NK*

Cell Phenotyping and Variability in Healthy Adults. Immunology Research 62 (3), 341–356.

[74] Lopez, C. F.; Muhlich, J. L.; Bachman, J. A.; Sorger, P. K. (2013) *Programming Biological Models in Python Using PySB.* Molecular Systems Biology 9, 646.

[90] Milo, R.; Jorgensen, P.; Moran, U.; Weber, G.; Springer, M. (2010). *BioNumbers—the database of key numbers in molecular and cell biology.* Nucleic Acids Research 38, D750–D753.

[99] StemCell Technologies (2019). *Frequencies of Cell Types in Human Peripheral Blood.* Document 23629, version 4.1.0.

[100] Sathaliyawala, T.; Kubota, M.; Yudanin, N.; Turner, D.; Camp, P.; Thome, J. J. C.; Bickham, K. L.; Lerner, H.; Goldstein, M.; Sykes, M.; Kato, T.; Farber, D. L. (2013) *Distribution and Compartmentalization of Human Circulating and Tissue-Resident Memory T Cell Subsets.* Immunity 38 (1), 187–197.

Appendix

For a system of 7 immune cell types (CD4 T cells, CD8 T cells, classical monocytes, non-classical monocytes, B cells, NK cells, dendritic cells), the corresponding full set of differential equations are:

$$\begin{aligned}
T4'(t) = {} & DC:T4(t) \cdot k_{T4:DC} + M14:T4(t) \cdot k_{T4:M14} + \\
& M16:T4(t) \cdot k_{T4:M16} + B:T4(t) \cdot k_{T4:B} + T4:T8(t) \cdot k_{T4:T8} + \\
& NK:T4(t) \cdot k_{T4:NK} + (0.5 \cdot T4(t)^2 \cdot k_{on}) \cdot (-2) + \\
& (T4:T4(t) \cdot k_{T4:T4}) \cdot 2 + (T4(t) \cdot T8(t) \cdot k_{on}) \cdot (-1) + \\
& (T4(t) \cdot NK(t) \cdot k_{on}) \cdot (-1) + (T4(t) \cdot DC(t) \cdot k_{on}) \cdot (-1) + \\
& (T4(t) \cdot M14(t) \cdot k_{on}) \cdot (-1) + (T4(t) \cdot M16(t) \cdot k_{on}) \cdot (-1) + \\
& (T4(t) \cdot B(t) \cdot k_{on}) \cdot (-1) \\
T8'(t) = {} & NK:T8(t) \cdot k_{T8:NK} + DC:T8(t) \cdot k_{T8:DC} + \\
& M14:T8(t) \cdot k_{T8:M14} + M16:T8(t) \cdot k_{T8:M16} + B:T8(t) \cdot k_{T8:B} + \\
& T4:T8(t) \cdot k_{T4:T8} + (0.5 \cdot T8(t)^2 \cdot k_{on}) \cdot (-2) + \\
& (T8:T8(t) \cdot k_{T8:T8}) \cdot 2 + (T4(t) \cdot T8(t) \cdot k_{on}) \cdot (-1) + \\
& (T8(t) \cdot NK(t) \cdot k_{on}) \cdot (-1) + (T8(t) \cdot DC(t) \cdot k_{on}) \cdot (-1) + \\
& (T8(t) \cdot M14(t) \cdot k_{on}) \cdot (-1) + (T8(t) \cdot M16(t) \cdot k_{on}) \cdot (-1) + \\
& (T8(t) \cdot B(t) \cdot k_{on}) \cdot (-1) \\
NK'(t) = {} & NK:T8(t) \cdot k_{T8:NK} + DC:NK(t) \cdot k_{NK:DC} + \\
& M14:NK(t) \cdot k_{NK:M14} + M16:NK(t) \cdot k_{NK:M16} + B:NK(t) \cdot k_{NK:B} + \\
& NK:T4(t) \cdot k_{T4:NK} + (0.5 \cdot NK(t)^2 \cdot k_{on}) \cdot (-2) + \\
& (NK:NK(t) \cdot k_{NK:NK}) \cdot 2 + (T4(t) \cdot NK(t) \cdot k_{on}) \cdot (-1) + \\
& (T8(t) \cdot NK(t) \cdot k_{on}) \cdot (-1) + (NK(t) \cdot DC(t) \cdot k_{on}) \cdot (-1) + \\
& (NK(t) \cdot M14(t) \cdot k_{on}) \cdot (-1) + (NK(t) \cdot M16(t) \cdot k_{on}) \cdot (-1) + \\
& (NK(t) \cdot B(t) \cdot k_{on}) \cdot (-1) \\
DC'(t) = {} & DC:T4(t) \cdot k_{T4:DC} + DC:T8(t) \cdot k_{T8:DC} + \\
& DC:NK(t) \cdot k_{NK:DC} + DC:M14(t) \cdot k_{DC:M14} + DC:M16(t) \cdot k_{DC:M16} + \\
& B:DC(t) \cdot k_{DC:B} + (DC:DC(t) \cdot k_{DC:DC}) \cdot 2 + \\
& (0.5 \cdot DC(t)^2 \cdot k_{on}) \cdot (-2) + (T4(t) \cdot DC(t) \cdot k_{on}) \cdot (-1) + \\
& (T8(t) \cdot DC(t) \cdot k_{on}) \cdot (-1) + (NK(t) \cdot DC(t) \cdot k_{on}) \cdot (-1) + \\
& (DC(t) \cdot M14(t) \cdot k_{on}) \cdot (-1) + (DC(t) \cdot M16(t) \cdot k_{on}) \cdot (-1) + \\
& (DC(t) \cdot B(t) \cdot k_{on}) \cdot (-1) \\
M14'(t) = {} & M14:T4(t) \cdot k_{T4:M14} + M14:T8(t) \cdot k_{T8:M14} + \\
& M14:NK(t) \cdot k_{NK:M14} + DC:M14(t) \cdot k_{DC:M14} + M14:M16(t) \cdot k_{M14:M16} + \\
& B:M14(t) \cdot k_{M14:B} + (M14:M14(t) \cdot k_{M14:M14}) \cdot 2 + \\
& (0.5 \cdot M14(t)^2 \cdot k_{on}) \cdot (-2) + (T4(t) \cdot M14(t) \cdot k_{on}) \cdot (-1) +
\end{aligned}$$

$$
\begin{aligned}
&(T8(t) \cdot M14(t) \cdot k_{on}) \cdot (-1) + (NK(t) \cdot M14(t) \cdot k_{on}) \cdot (-1)+\\
&(DC(t) \cdot M14(t) \cdot k_{on}) \cdot (-1) + (M14(t) \cdot M16(t) \cdot k_{on}) \cdot (-1)+\\
&(M14(t) \cdot B(t) \cdot k_{on}) \cdot (-1)\\
M16'(t) &= M16 : T4(t) \cdot k_{T4:M16} + M16 : T8(t) \cdot k_{T8:M16}+\\
&M16 : NK(t) \cdot k_{NK:M16} + DC : M16(t) \cdot k_{DC:M16} + M14 : M16(t) \cdot k_{M14:M16}+\\
&B : M16(t) \cdot k_{M16:B} + (M16 : M16(t) \cdot k_{M16:M16}) \cdot 2+\\
&(0.5 \cdot M16(t)^2 \cdot k_{on}) \cdot (-2) + (T4(t) \cdot M16(t) \cdot k_{on}) \cdot (-1)+\\
&(T8(t) \cdot M16(t) \cdot k_{on}) \cdot (-1) + (NK(t) \cdot M16(t) \cdot k_{on}) \cdot (-1)+\\
&(DC(t) \cdot M16(t) \cdot k_{on}) \cdot (-1) + (M14(t) \cdot M16(t) \cdot k_{on}) \cdot (-1)+\\
&(M16(t) \cdot B(t) \cdot k_{on}) \cdot (-1)\\
B'(t) &= B : T4(t) \cdot k_{T4:B} + B : T8(t) \cdot k_{T8:B} + B : NK(t) \cdot k_{NK:B}+\\
&B : DC(t) \cdot k_{DC:B} + B : M14(t) \cdot k_{M14:B} + B : M16(t) \cdot k_{M16:B}+\\
&(B : B(t) \cdot k_{B:B}) \cdot 2 + (0.5 \cdot B(t)^2 \cdot k_{on}) \cdot (-2)+\\
&(T4(t) \cdot B(t) \cdot k_{on}) \cdot (-1) + (T8(t) \cdot B(t) \cdot k_{on}) \cdot (-1)+\\
&(NK(t) \cdot B(t) \cdot k_{on}) \cdot (-1) + (DC(t) \cdot B(t) \cdot k_{on}) \cdot (-1)+\\
&(M14(t) \cdot B(t) \cdot k_{on}) \cdot (-1) + (M16(t) \cdot B(t) \cdot k_{on}) \cdot (-1)\\
T4 : T4'(t) &= 0.5 \cdot T4(t)^2 \cdot k_{on} + (T4 : T4(t) \cdot k_{T4:T4}) \cdot (-1)\\
T4 : T8'(t) &= T4(t) \cdot T8(t) \cdot k_{on} + (T4 : T8(t) \cdot k_{T4:T8}) \cdot (-1)\\
NK : T4'(t) &= T4(t) \cdot NK(t) \cdot k_{on} + (NK : T4(t) \cdot k_{T4:NK}) \cdot (-1)\\
DC : T4'(t) &= T4(t) \cdot DC(t) \cdot k_{on} + (DC : T4(t) \cdot k_{T4:DC}) \cdot (-1)\\
M14 : T4'(t) &= T4(t) \cdot M14(t) \cdot k_{on} + (M14 : T4(t) \cdot k_{T4:M14}) \cdot (-1)\\
M16 : T4'(t) &= T4(t) \cdot M16(t) \cdot k_{on} + (M16 : T4(t) \cdot k_{T4:M16}) \cdot (-1)\\
B : T4'(t) &= T4(t) \cdot B(t) \cdot k_{on} + (B : T4(t) \cdot k_{T4:B}) \cdot (-1)\\
T8 : T8'(t) &= 0.5 \cdot T8(t)^2 \cdot k_{on} + (T8 : T8(t) \cdot k_{T8:T8}) \cdot (-1)\\
NK : T8'(t) &= T8(t) \cdot NK(t) \cdot k_{on} + (NK : T8(t) \cdot k_{T8:NK}) \cdot (-1)\\
DC : T8'(t) &= T8(t) \cdot DC(t) \cdot k_{on} + (DC : T8(t) \cdot k_{T8:DC}) \cdot (-1)\\
M14 : T8'(t) &= T8(t) \cdot M14(t) \cdot k_{on} + (M14 : T8(t) \cdot k_{T8:M14}) \cdot (-1)\\
M16 : T8'(t) &= T8(t) \cdot M16(t) \cdot k_{on} + (M16 : T8(t) \cdot k_{T8:M16}) \cdot (-1)\\
B : T8'(t) &= T8(t) \cdot B(t) \cdot k_{on} + (B : T8(t) \cdot k_{T8:B}) \cdot (-1)\\
NK : NK'(t) &= 0.5 \cdot NK(t)^2 \cdot k_{on} + (NK : NK(t) \cdot k_{NK:NK}) \cdot (-1)\\
DC : NK'(t) &= NK(t) \cdot DC(t) \cdot k_{on} + (DC : NK(t) \cdot k_{NK:DC}) \cdot (-1)\\
M14 : NK'(t) &= NK(t) \cdot M14(t) \cdot k_{on} + (M14 : NK(t) \cdot k_{NK:M14}) \cdot (-1)\\
M16 : NK'(t) &= NK(t) \cdot M16(t) \cdot k_{on} + (M16 : NK(t) \cdot k_{NK:M16}) \cdot (-1)\\
B : NK'(t) &= NK(t) \cdot B(t) \cdot k_{on} + (B : NK(t) \cdot k_{NK:B}) \cdot (-1)\\
DC : DC'(t) &= 0.5 \cdot DC(t)^2 \cdot k_{on} + (DC : DC(t) \cdot k_{DC:DC}) \cdot (-1)\\
DC : M14'(t) &= DC(t) \cdot M14(t) \cdot k_{on} + (DC : M14(t) \cdot k_{DC:M14}) \cdot (-1)
\end{aligned}
$$

$$
\begin{aligned}
DC:M16'(t) &= DC(t)\cdot M16(t)\cdot k_{on} + (DC:M16(t)\cdot k_{DC:M16})\cdot(-1)\\
B:DC'(t) &= DC(t)\cdot B(t)\cdot k_{on} + (B:DC(t)\cdot k_{DC:B})\cdot(-1)\\
M14:M14'(t) &= 0.5\cdot M14(t)^2\cdot k_{on} + (M14:M14(t)\cdot k_{M14:M14})\cdot(-1)\\
M14:M16'(t) &= M14(t)\cdot M16(t)\cdot k_{on} + (M14:M16(t)\cdot k_{M14:M16})\cdot(-1)\\
B:M14'(t) &= M14(t)\cdot B(t)\cdot k_{on} + (B:M14(t)\cdot k_{M14:B})\cdot(-1)\\
M16:M16'(t) &= 0.5\cdot M16(t)^2\cdot k_{on} + (M16:M16(t)\cdot k_{M16:M16})\cdot(-1)\\
B:M16'(t) &= M16(t)\cdot B(t)\cdot k_{on} + (B:M16(t)\cdot k_{M16:B})\cdot(-1)\\
B:B'(t) &= 0.5\cdot B(t)^2\cdot k_{on} + (B:B(t)\cdot k_{B:B})\cdot(-1)
\end{aligned}
$$
