# Supplementary material for: A physical wiring diagram for the human immune system
Source: Nature. 2022 Aug 3;608(7922):397–404. doi: 10.1038/s41586-022-05028-x (PMC9365698; doi:10.1038/s41586-022-05028-x)
Supplement: Supplementary file 2 — Reporting Summary [file 41586_2022_5028_MOESM2_ESM.pdf]

nature portfolio

Corresponding author(s): Jarrod Shilts, Gavin J. Wright

Last updated by author(s): Apr 18, 2022

Reporting Summary

Nature Portfolio wishes to improve the reproducibility of the work that we publish. This form provides structure for consistency and transparency in reporting. For further information on Nature Portfolio policies, see our Editorial Policies and the Editorial Policy Checklist.

Statistics

For all statistical analyses, confirm that the following items are present in the figure legend, table legend, main text, or Methods section.

n/a	Confirmed	
☐	☒	The exact sample size (*n*) for each experimental group/condition, given as a discrete number and unit of measurement
☐	☒	A statement on whether measurements were taken from distinct samples or whether the same sample was measured repeatedly
☐	☒	The statistical test(s) used AND whether they are one- or two-sided<br>*Only common tests should be described solely by name; describe more complex techniques in the Methods section.*
☒	☐	A description of all covariates tested
☐	☒	A description of any assumptions or corrections, such as tests of normality and adjustment for multiple comparisons
☐	☒	A full description of the statistical parameters including central tendency (e.g. means) or other basic estimates (e.g. regression coefficient) AND variation (e.g. standard deviation) or associated estimates of uncertainty (e.g. confidence intervals)
☐	☒	For null hypothesis testing, the test statistic (e.g. *F*, *t*, *r*) with confidence intervals, effect sizes, degrees of freedom and *P* value noted<br>*Give P values as exact values whenever suitable.*
☒	☐	For Bayesian analysis, information on the choice of priors and Markov chain Monte Carlo settings
☒	☐	For hierarchical and complex designs, identification of the appropriate level for tests and full reporting of outcomes
☒	☐	Estimates of effect sizes (e.g. Cohen's *d*, Pearson's *r*), indicating how they were calculated

Our web collection on statistics for biologists contains articles on many of the points above.

Software and code

Policy information about availability of computer code

Data collection	Optical absorbance measurements were collected on a Tecan Spark plate reader. Surface plasmon resonance data were collected on a BIAcore 8k instrument. Flow cytometry data were collected on Becton-Dickinson LSR Fortessa flow cytometer. Microscopy images were taken using a Perkin Elmer Opera Phenix confocal microscope.
Data analysis	All custom analysis code is written in R and Python, which are open projects without license restrictions. R is version 4.0.3 and Python is version 3.7.4. The following packages were also used. For R: Seurat (version 3.1.5), shinyCircos (version 1.0.0), PRROC (version 1.3.1), NicheNet (version 1.0.0), DEseq2 (version 1.30.0), igraph (version 1.2.5). For Python: Scanpy (version 1.4.5), PySB (version 1.11). FlowJo (version 10.6.1) was used for drawing gates on flow cytometry data. For microscopy image analysis, both CellProfiler (version 2) and MATLAB Neural Network Toolbox (version R2020a) were used.

For manuscripts utilizing custom algorithms or software that are central to the research but not yet described in published literature, software must be made available to editors and reviewers. We strongly encourage code deposition in a community repository (e.g. GitHub). See the Nature Portfolio guidelines for submitting code & software for further information.

Data

Policy information about availability of data

All manuscripts must include a data availability statement. This statement should provide the following information, where applicable:

- Accession codes, unique identifiers, or web links for publicly available datasets
- A description of any restrictions on data availability
- For clinical datasets or third party data, please ensure that the statement adheres to our policy

All data files are available in the Github repository associated with this manuscript (https://github.com/jshilts/shilts-et-al-2022-immunoreceptors). Accession codes are provided by the UniProt database (https://uniprot.org/).

Field-specific reporting

Please select the one below that is the best fit for your research. If you are not sure, read the appropriate sections before making your selection.

☒ Life sciences ☐ Behavioural & social sciences ☐ Ecological, evolutionary & environmental sciences

For a reference copy of the document with all sections, see nature.com/documents/nr-reporting-summary-flat.pdf

Life sciences study design

All studies must disclose on these points even when the disclosure is negative.

Sample size	For automated microscopy experiments, a sample size of 5 per concentration per protein condition was selected as the largest number of replicates that could be fit onto the screening plates run for the platform. This number is equal to or greater than sample sizes previously published as sufficient for this method, e.g. DOI 10.1038/NCHEMBIO.2360. For binding screens, two replicates for each bait-prey orientation were done based on calculations for ensuring any rare false-positive signals on an individual replicate could be corrected, as informed by our earlier false positive rate benchmarking.
Data exclusions	No binding screen measurements were omitted. For specific analyses such as measuring protein-protein interaction signals from screening independent of known carbohydrate-binding lectin receptors, the criteria used for defining that excluded group are explained in the methods. Automated microscopy microscopy measurements of non-classical monocytes were excluded because so few cells were detected that they were not possible to analyze (e.g. cell counts jumping between 0 and 1 resulting in undefined fold-changes).
Replication	All of the novel interactions we describe in our study have been replicated by multiple independent approaches, as described in the text and summarized in the Extended Data figures. Binding assays measured two separate orientations for each protein, with two of these complete binding assays being done for every interaction. Every positive signal identified was then verified by binding onto human cell lines and through biophysical approaches verifying the binding was saturable and measuring the kinetics of binding.
Randomization	The order of all proteins in the systematic binding assays were randomized by a computer before screening. The positions of every condition in the automated microscopy experiment was also randomized on each plate.
Blinding	Investigators were blinded to the identities of samples during interaction screening. For other experiments such as surface plasmon resonance measurements, the samples could not be blinded.

Reporting for specific materials, systems and methods

We require information from authors about some types of materials, experimental systems and methods used in many studies. Here, indicate whether each material, system or method listed is relevant to your study. If you are not sure if a list item applies to your research, read the appropriate section before selecting a response.

Materials & experimental systems

n/a	Involved in the study	
☐	☒	Antibodies
☐	☒	Eukaryotic cell lines
☒	☐	Palaeontology and archaeology
☒	☐	Animals and other organisms
☐	☒	Human research participants
☒	☐	Clinical data
☒	☐	Dual use research of concern

Methods

n/a	Involved in the study	
☒	☐	ChIP-seq
☐	☒	Flow cytometry
☒	☐	MRI-based neuroimaging

Antibodies

Antibodies used	For leukocyte immunostaining, the following antibodies raised against human epitopes were used: anti-CD3 AF647 (Biolegend, Clone UCHT1, cat. #300416, lot #B284504), anti-CD4 FITC (Biolegend, Clone SK3, cat. #344604, lot #B244280), anti-CD8 PE (BD Biosciences, Clone SK1, cat. #345773, lot #106349), anti-CD19 FITC (Biolegend, Clone SJ25C1, cat. #363008, lot #B290869), anti-CD56 PE (Beckman Coulter, Clone N901, cat. #A07788, lot #49), anti-CD16 PE (Biolegend, Clone 3G8, cat. #302008, lot #B290852), anti-CD14 AF647 (Biolegend, Clone HCD14, cat. #325612, lot #B260484), anti-CD20 (BD Biosciences, Clone 2H7, cat. #555623, lot #8260745). For protein normalization ELISAs, the following antibodies were used: anti-rat Cd4 (Clone OX68, purified from a hybridoma provided by Neil Barclay, University of Oxford), and anti-mouse IgG alkaline phosphatase (Sigma, cat. #A9316).
Validation	Every antibody used was validated for specificity to their human cell-surface targets by their manufacturers, and all pharmacoscopy antibodies have been established for use as a panel by a previous study (Severin et al., 10.1101/2021.12.03.471105). The antibodies against CD3, CD8, CD14, and CD20 were also validated by their manufacturers for immunocytochemistry, while all remaining antibodies were validated by their manufactures for flow cytometry. The OX68 antibody has further had its specificity validated by prior studies (e.g. 10.1186/1471-2091-6-2).

Eukaryotic cell lines

Policy information about cell lines

Cell line source(s)	Both HEK293 cell lines were graciously provided by Yves Durocher (National Research Council, Canada).
Authentication	HEK293 cell lines were not authenticated before this study.
Mycoplasma contamination	All cell lines were regularly tested for mycoplasma (Surrey Diagnostics, UK) and found to be negative all throughout these experiments.
Commonly misidentified lines (See ICLAC register)	Our cell lines are not listed as commonly misidentified.

Human research participants

Policy information about studies involving human research participants

Population characteristics	Blood donors were healthy Swiss residents, with hemoglobin levels and blood pressure readings within normal reference ranges. The characteristics of the deceased organ donors for lymph node specimens were not recorded.
Recruitment	Anonymous organ donors were identified by the Cambridge Biorepository for Translational Medicine (CBTM). Blood donations were collected by Blutspende Zürich.
Ethics oversight	Tissue collection was overseen by the Cambridge Biorepository for Translational Medicine (CBTM) with full approval from the National Research Ethics Service Committee East of England - Cambridge South (15/EE/0152). CBTM operates in accordance with UK Human Tissue Authority guidelines. All tissues were taken from deceased organ transplant donors with informed consent from the donor families. Blood collection was overseen by the cantonal ethical committee of Zurich (KEK Zurich, BASEC-Nr 2019-01579) with consent from the blood donors.

Note that full information on the approval of the study protocol must also be provided in the manuscript.

Flow Cytometry

Plots

Confirm that:

☒ The axis labels state the marker and fluorochrome used (e.g. CD4-FITC).

☒ The axis scales are clearly visible. Include numbers along axes only for bottom left plot of group (a 'group' is an analysis of identical markers).

☒ All plots are contour plots with outliers or pseudocolor plots.

☒ A numerical value for number of cells or percentage (with statistics) is provided.

Methodology

Sample preparation	HEK293 cells were incubated with fluorescent tetrameters loaded with a recombinant protein extracellular domain. Only a single fluorochrome was use d, hence some of the points listed above are not applicable.
Instrument	BD LSRFortessa Flow Cytometer
Software	FlowJo (version 10.6.1) was used for drawing gates. The R package ggcyto (version 1.17.0) was used for drawing histogram plots of the gated data.

Cell population abundance	No sorting was performed. All cells began as homogeneous HEK293 cultures, ensuring their purity. At least 10,000 cells were measured for each condition.
Gating strategy	Cell-sized events were gated based on SSC-A / FSC-A as shown in the supplementary example gates. Singlet cells were selected based on FSC-W / FSC-A.

☒ Tick this box to confirm that a figure exemplifying the gating strategy is provided in the Supplementary Information.
