# Supplementary material for: A physical wiring diagram for the human immune system
Source: Nature. 2022 Aug 3;608(7922):397–404. doi: 10.1038/s41586-022-05028-x (PMC9365698; doi:10.1038/s41586-022-05028-x)
Supplement: Supplementary file 3 — Supplementary Figs. 1–4 show the complete uncropped images of Coomassie-stained protein gels and protein gel filtration traces. Example images illustrating the flow cytometry gating strategy are also included. [file 41586_2022_5028_MOESM3_ESM.pdf]

Supplementary images for

"A physical wiring diagram for the human immune system"

Jarrod Shilts, Yannik Severin, Francis Galaway, Nicole Müller-Sienerth,
Zheng-Shan Chong, Sophie Pritchard, Sarah Teichmann,
Roser Vento-Tormo, Berend Snijder, and Gavin J. Wright

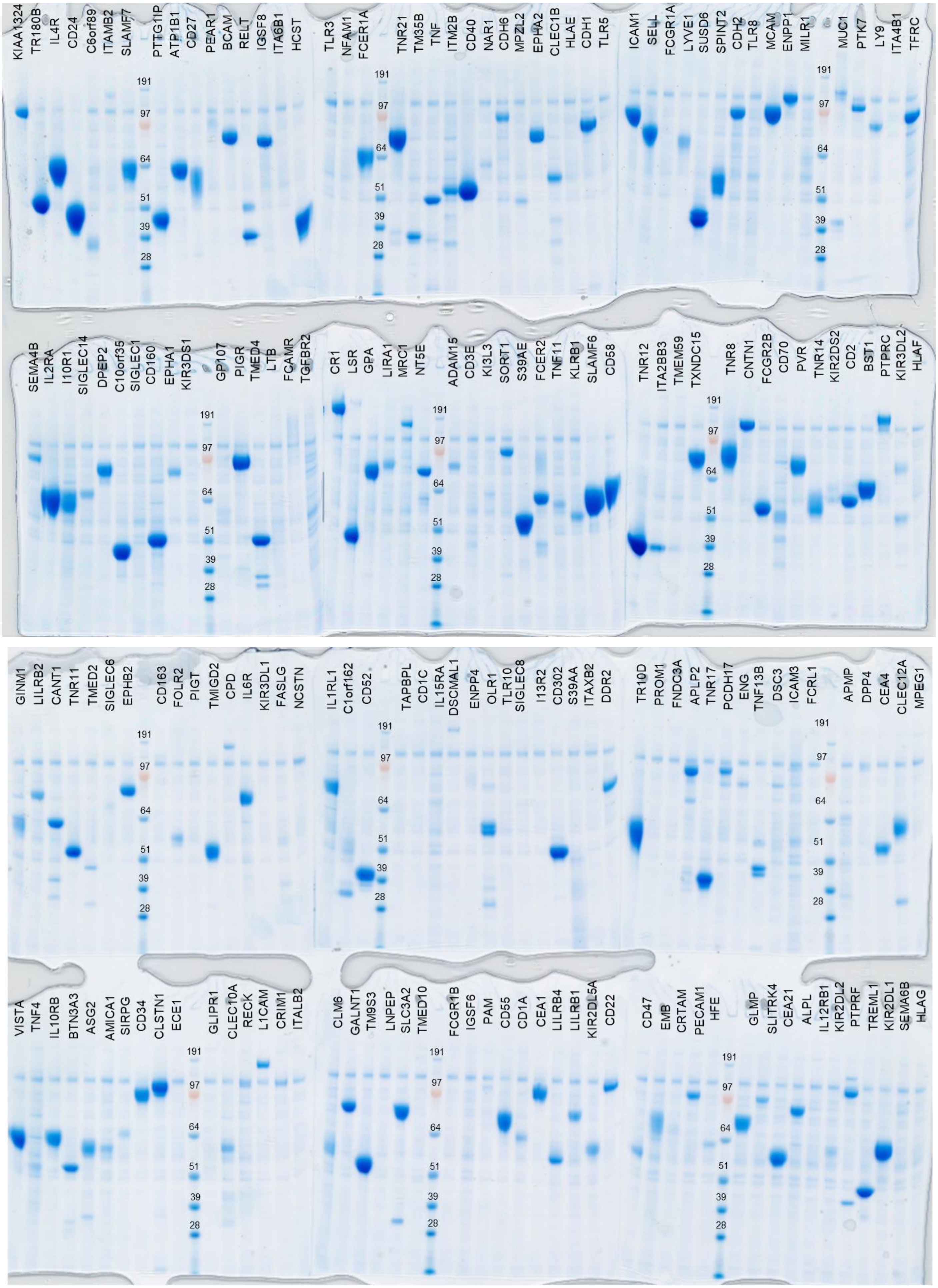
KIAA1324 TR180B IL4R CD24 C6orf89 ITAMB2 SLAMF7 PTTG1IP ATP1B1 CD27 PEAR1 BCAM RELT IGSF8 ITA6B1 HCST
TLR3 NFAM1 FCER1A TNR21 TM35B TNF ITM2B CD40 NAR1 CDH6 MPZL2 EPHA2 CLEC1B HLAE CDH1 TLR5
ICAM1 SELL FCGR1A LYVE1 SUSD6 SPINT2 CDH2 TLR8 MCAM ENPP1 MILR1 MUC1 PTK7 LY9 ITA4B1 TFRC
SEMA4B IL2RA I10R1 SIGLEC14 DPEP2 C10orf35 SIGLEC1 CD160 EPHA1 KIR3DS1 GP107 PIGR TMED4 LTB FCAMR TGFBR2
CR1 LSR GPA LIRA1 MRC1 NT5E ADAM15 CD3E KI3L3 SORT1 S39AE FCER2 TNF11 KLRB1 SLAMF6 CD58
TNR12 ITA2BB3 TMEM59 TXNDC15 TNR8 CNTN1 FCGR2B CD70 PVR TNR14 KIR2DS2 CD2 BST1 PTPRC KIR3DL2 HLAF
GINM1 LILRB2 CANT1 TNR11 TMED2 SIGLEC6 EPHB2 CD163 FOLR2 PIGT TMIGD2 CPD IL6R KIR3DL1 FASLG NCSTN
IL1RL1 C1orf162 CD52 TAPBPL CD1C IL15RA DSCMAL1 ENPP4 OLR1 TLR10 SIGLEC8 I13R2 CD302 S39AA ITAXB2 DDR2
TR10D PROM1 FNDC3A APLP2 TNR17 PCDH17 ENG TNF13B DSC3 ICAM3 FCRL1 APMP DPP4 CEA4 CLEC12A MPEG1
VISTA TNF4 IL10RB BTN3A3 ASG2 AMICA1 SIRPG CD34 CLSTN1 ECE1 GLIPR1 CLEC10A RECK L1CAM CRIM1 ITALB2
CLM6 GALNT1 TM9S3 LNPEP SLC3A2 TMED10 FCGR1B IGSF6 PAM CD55 CD1A CEA1 LILRB4 LILRB1 KIR2DL5A CD22
CD47 EMB CRTAM PECAM1 HFE GLMP SLITRK4 CEA21 ALPL IL12RB1 KIR2DL2 PTPRF TREML1 KIR2DL1 SEMA6B HLAG
191
97
64
51
39
28

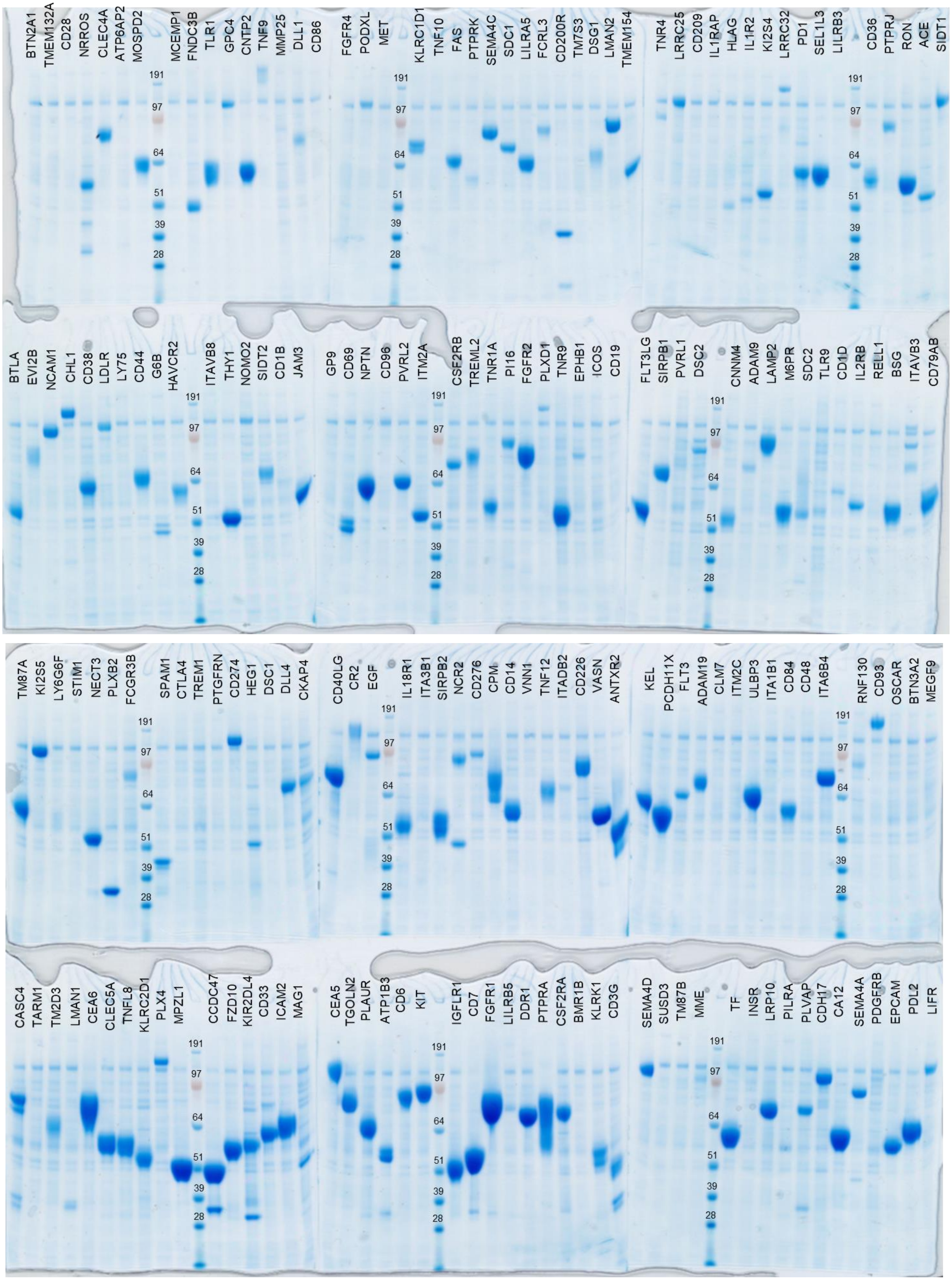
BTN2A1
TMEM132A
CD28
NRROS
CLEC4A
ATP6AP2
MOSPD2
191 97 64 51 39 28
MCEMP1
FNDC3B
TLR1
GPC4
CNTP2
TNF9
MMP25
DLL1
CD86
FGFR4
PODXL
MET
191 97 64 51 39 28
KLRC1D1
TNF10
FAS
PTPRK
SEMA4C
SDC1
LILRA5
FCRL3
CD200R
TM7S3
DSG1
LMAN2
TMEM154
TNR4
LRRC25
CD209
IL1RAP
HLAG
IL1R2
KI2S4
LRRC32
PD1
SEL1L3
LILRB3
191 97 64 51 39 28
CD36
PTPRJ
RON
ACE
SIDT1
BTLA
EVI2B
NCAM1
CHL1
CD38
LDLR
LY75
CD44
G6B
HAVCR2
191 97 64 51 39 28
ITAVB8
THY1
NOMO2
SIDT2
CD1B
JAM3
GP9
CD69
NPTN
CD96
PVRL2
ITM2A
191 97 64 51 39 28
CSF2RB
TREML2
TNR1A
PI16
FGFR2
PLXD1
TNR9
EPHB1
ICOS
CD19
FLT3LG
SIRPB1
PVRL1
DSC2
191 97 64 51 39 28
CNNM4
ADAM9
LAMP2
M6PR
SDC2
TLR9
CD1D
IL2RB
RELL1
BSG
ITAVB3
CD79AB
TM87A
KI2S5
LY6G6F
STIM1
NECT3
PLXB2
FCGR3B
191 97 64 51 39 28
SPAM1
CTLA4
TREM1
PTGFRN
CD274
HEG1
DSC1
DLL4
CKAP4
CD40LG
CR2
EGF
191 97 64 51 39 28
IL18R1
ITA3B1
SIRPB2
NCR2
CD276
CPM
CD14
VNN1
TNF12
ITADB2
CD226
VASN
ANTXR2
KEL
PCDH11X
FLT3
ADAM19
CLM7
ITM2C
ULBP3
ITA1B1
CD84
CD48
ITA6B4
191 97 64 51 39 28
RNF130
CD93
OSCAR
BTN3A2
MEGF9
CASC4
TARM1
TM2D3
LMAN1
CEA6
CLEC5A
TNFL8
KLRC2D1
PLX4
MPZL1
191 97 64 51 39 28
CCDC47
FZD10
KIR2DL4
CD33
ICAM2
MAG1
CEA5
TGOLN2
PLAUR
ATP1B3
CD6
KIT
191 97 64 51 39 28
IGFLR1
CD7
FGFR1
LILRB5
DDR1
PTPRA
CSF2RA
BMR1B
KLRK1
CD3G
SEMA4D
SUSD3
TM87B
MME
191 97 64 51 39 28
TF
INSR
LRP10
PILRA
PLVAP
CDH17
CA12
SEMA4A
PDGFRB
EPCAM
PDL2
LIFR

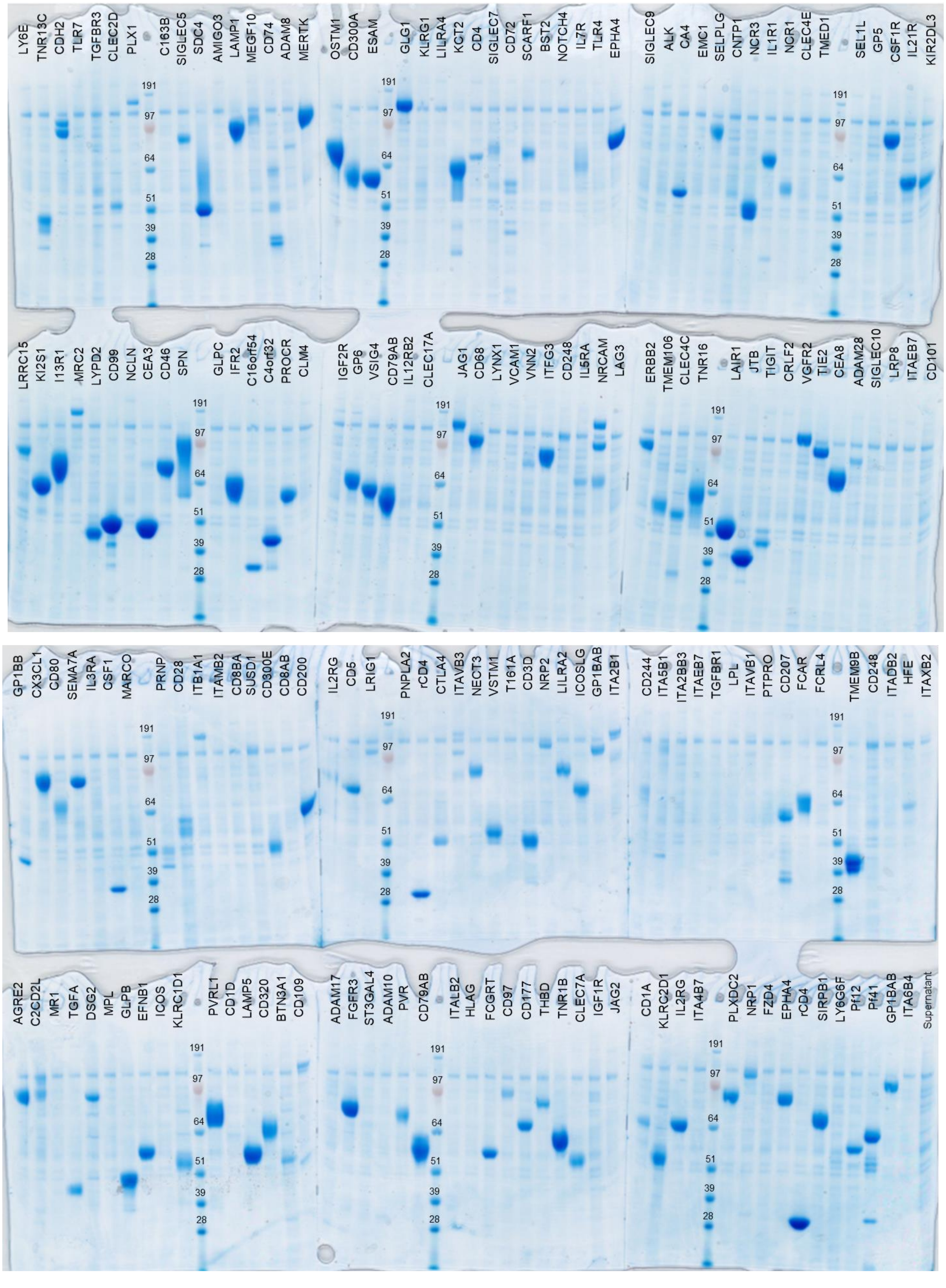
LY6E TNR13C CDH2 TLR7 TGFBR3 CLEC2D PLX1 C163B SIGLEC5 SDC4 AMIGO3 LAMP1 MEGF10 CD74 ADAM8 MERTK
OSTM1 CD300A ESAM GLG1 KLRG1 LILRA4 KCT2 CD4 SIGLEC7 CD72 SCARF1 BST2 NOTCH4 IL7R TLR4 EPHA4
SIGLEC9 ALK CA4 EMC1 SELPLG CNTP1 NCR3 IL1R1 NCR1 CLEC4E TMED1 SEL1L GP5 CSF1R IL21R KIR2DL3
LRRC15 KI2S1 I13R1 MRC2 LYPD2 CD99 NCLN CEA3 CD46 SPN GLPC IFR2 C16orf54 C4orf32 PROCR CLM4
IGF2R GP6 VSIG4 CD79AB IL12RB2 CLEC17A JAG1 CD68 LYNX1 VCAM1 VNN2 ITFG3 CD248 IL5RA NRCAM LAG3
ERBB2 TMEM106 CLEC4C TNR16 LAIR1 JTB TIGIT CRLF2 VGFR2 TIE2 CEA8 ADAM28 SIGLEC10 LRP8 ITAEB7 CD101
GP1BB CX3CL1 CD80 SEMA7A IL3RA CSF1 MARCO PRNP CD28 ITB1A1 ITAMB2 CD8BA SUSD1 CD300E CD8AB CD200
IL2RG CD5 LRIG1 PNPLA2 rCD4 CTLA4 ITAVB3 NECT3 VSTM1 T161A CD3D NRP2 LILRA2 ICOSLG GP1BAB ITA2B1
CD244 ITA5B1 ITA2BB3 ITAEB7 TGFBR1 LPL ITAVB1 PTPRO CD207 FCAR FCRL4 TMEM9B CD248 ITADB2 HFE ITAXB2
AGRE2 C2CD2L MR1 TGFA DSG2 MPL GLPB EFNB1 ICOS KLRC1D1 PVRL1 CD1D LAMP5 CD320 BTN3A1 CD109
ADAM17 FGFR3 ST3GAL4 ADAM10 PVR CD79AB ITALB2 HLAG FCGRT CD97 CD177 THBD TNR1B CLEC7A IGF1R JAG2
CD1A KLRC2D1 IL2RG ITA4B7 PLXDC2 NRP1 FZD4 EPHA4 rCD4 SIRPB1 LY6G6F Pf12 Pf41 GP1BAB ITA6B4 Supernatant
191
97
64
51
39
28

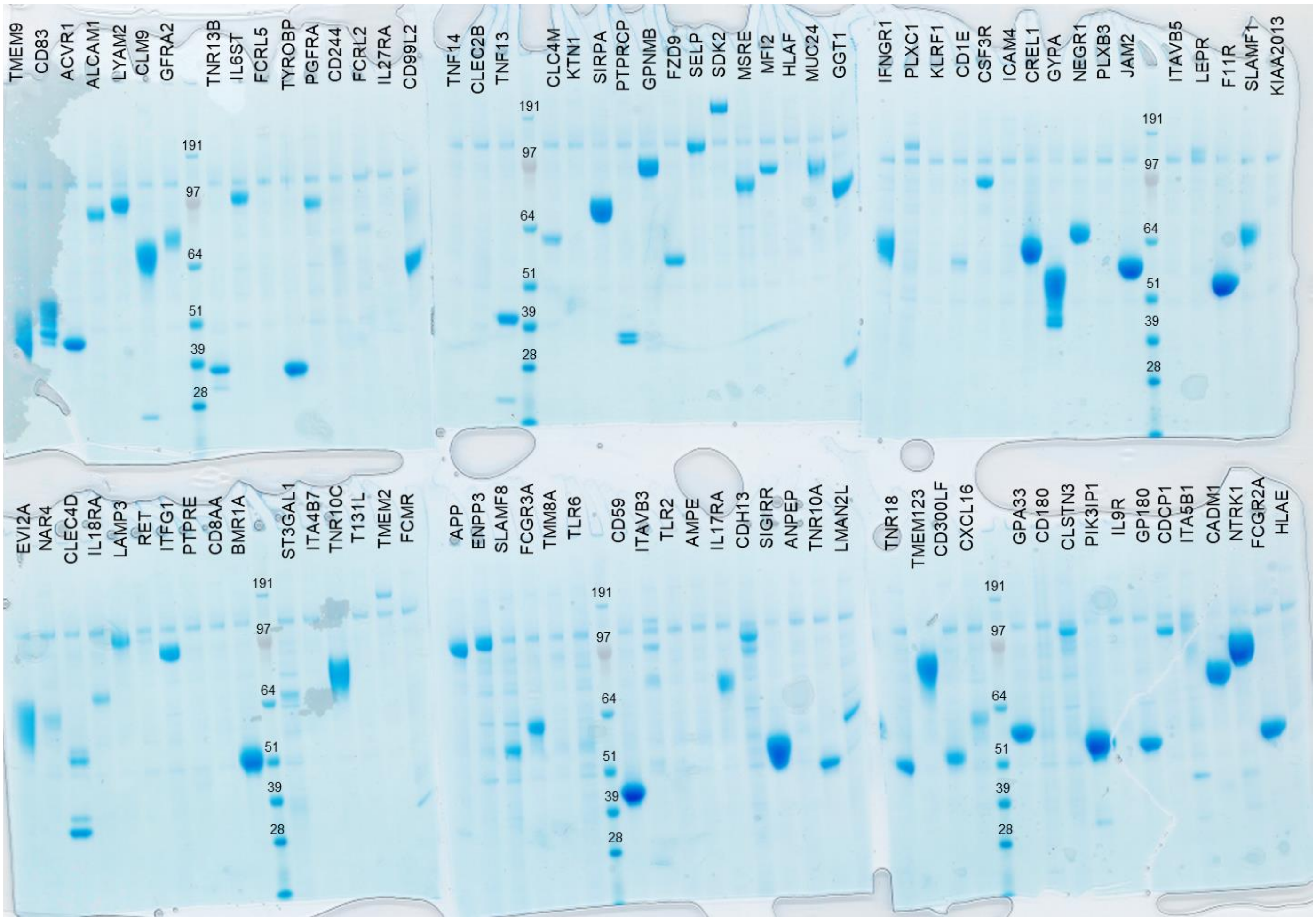

Supplementary Figure 1. Full purified recombinant protein library of immune receptors. Total protein content detectable by Coomassie staining of all samples post-purification. The molecular masses in kilodaltons are indicated on each standard marker. The recurring non-target band around 100 kDa is serum protein.

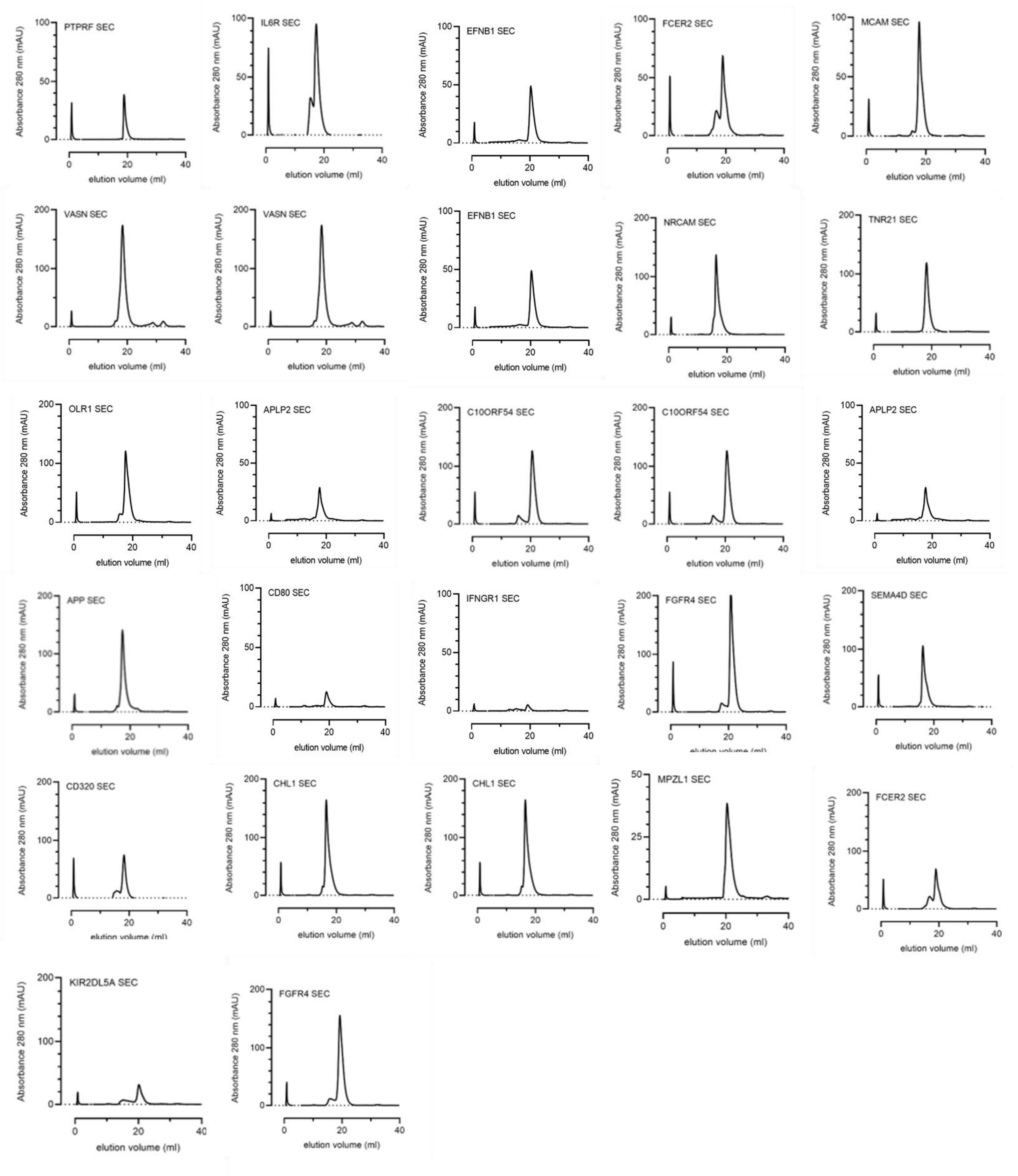

Supplementary Figure 2. Full size exclusion chromatography traces of surface plasmon resonance analytes. Panels are arranged to match Extended Data Figure 4B, including duplicating analytes which were used for multiple surface plasmon resonance experiments. The fraction corresponding to the monomeric protein was collected for all experiments.

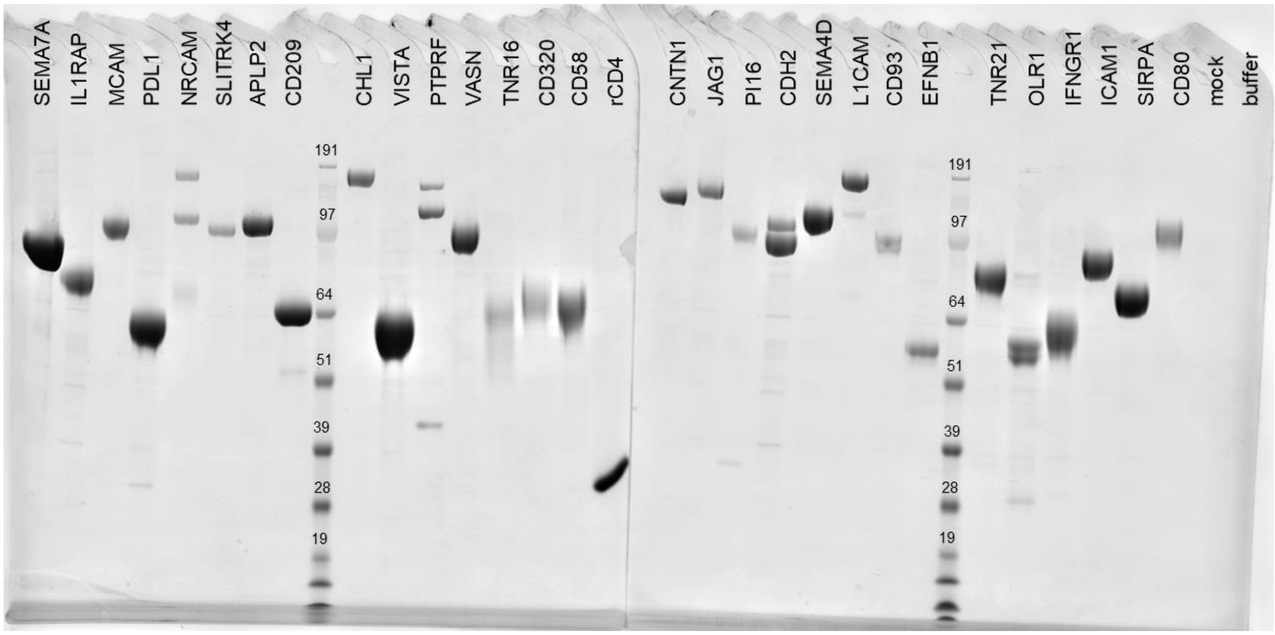

Supplementary Figure 3. Full set of high-purity recombinant proteins applied to human leukocytes for high-content microscopy assays. Coomassie staining of total protein following high-stringency purification and dialysis. The molecular masses in kilodaltons are indicated on each standard marker.

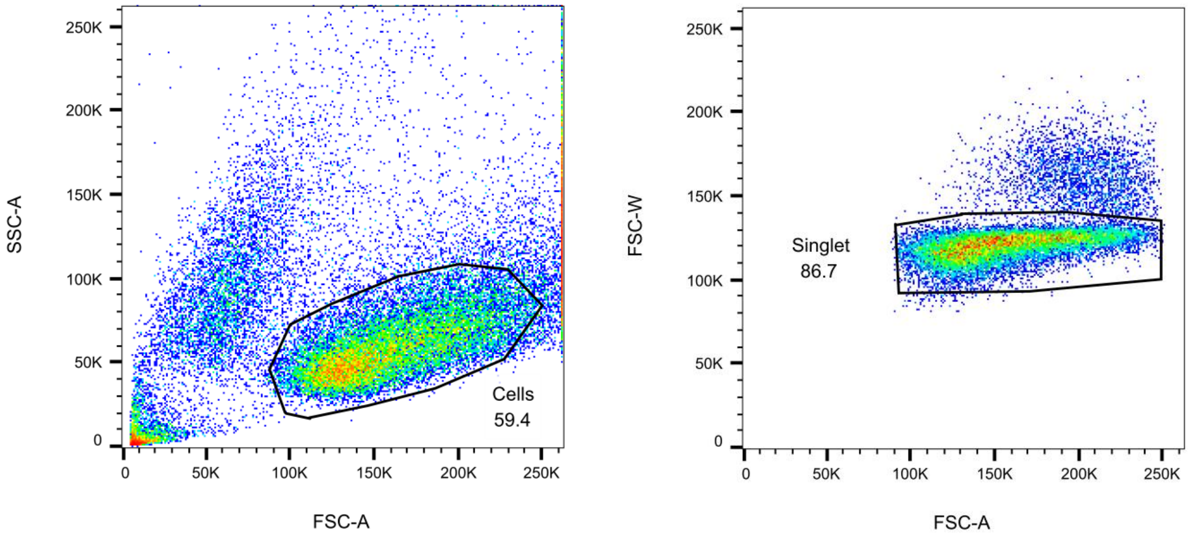

Supplementary Figure 4. Gating strategy example.
Example of the flow-cytometry gating strategy for measuring single HEK293 cells. The “Singlet” gate is a subset of the “Cells” gate. Percentages of cells within the gate are shown below each gate’s name.
